# A London Fever-hole

**Published:** 1854-10

**Authors:** 


*A London Fever-hole.*—A correspondent to a daily paper, (*Daily News*, Aug. 16th,) in alluding to the low parts of St. Clements Danes as

"London fever hole," thus draws the abode of many Christian men and women of "Merrie England :"—

"A track through the heart of the Black Forest, or a pass through the bowels of a mountain in Arabia Petrea, could not be more close and gloomy. You might walk here in a good stiff hurricane, and hardly know it; a summer shower might pass, and leave you dry. You are in the region of perpetual shadow; and the women and children who sit or sprawl upon the door-steps are scarcely less in doors than when languishing in their dark and fetid rooms. And no wonder; for, according to actual measurement, the courts vary in breadth from six to twelve feet. Here are the holes in which our human fellow-creatures swarm like vermin. According to a report published in the *Daily News* of May 1st, no less than fifty inmates were found to reside in one of the houses in Middle Serle's-place, (formerly Little Shire-lane;) and in Ship-yard many of the houses are built back to back, entirely preventing thorough ventilation. The gentlemen who made the examination state that water-butts are kept in underground cellars, the walls and flooring of which are continually damp to the touch, and where the water, imbibing the filthy exhalations of the place, acquires a dreadful odor; that the ceilings of some of these cellars are actually below the level of the roadways, so that the inhabitants are obliged to burn candles through the whole day, with the exception of a few hours, and that terrier dogs are kept in many of the houses as a protection against the rats. Yet out of these hideous tenements considerable sums of money are drawn every year by letting and sub-letting. Hideous women, foul and slatternly, loll out of windows or lean against door-posts, overcome with the terrible lassitude and indolence which cannot fail to arise from the influences by which they are surrounded; not impudent and brazen, but oppressed with the hopeless burden of their lives. The children, sullen, dirty, and fierce,—young tigers without their beauty or their health,—play or fight in the roadways, amidst cabbage-stalks, potato-peelings, oyster-shells, and standing puddles. Men are very seldom seen. And over the young and the old tower the melancholy house fronts, shutting out the sky and the breeze, and black and saturated with the pestilent vapors which, rising unseen around them,

Hang their poison
In the sick air."

*Lancet.*

